# How Are TBI Symptoms Interconnected? A Network Analysis Approach

**DOI:** 10.1002/brb3.70316

**Published:** 2025-02-16

**Authors:** Helen Bindels, Sascha Sommer, Tobias Ohmann, Susann Seddigh, Michael Schuler

**Affiliations:** ^1^ Department of Nursing, Midwifery and Therapy Sciences Bochum University of Applied Sciences Bochum Germany; ^2^ Research Department BG Klinikum Duisburg Duisburg Germany

**Keywords:** long‐term sequelae, symptom networks, traumatic brain injury

## Abstract

**Background:**

Many patients with traumatic brain injury (TBI) across all levels of severity experience persistent psycho‐emotional, cognitive, and somatic symptoms. Psychological network theory views disorders as intricate systems rather than discrete diseases. This study employs an exploratory network analysis method to uncover potential causal links among long‐term TBI symptoms.

**Methods:**

We examined persistent symptoms using secondary data from 250 TBI patients undergoing an inpatient “brain check” procedure. We constructed two partial correlation networks: one for the entire sample and another for a mild TBI subgroup, each consisting of 14 symptoms and three covariates. The symptoms and their connections were visualized in network graphs to identify potential causal, and structural indicators and centrality indices were calculated.

**Results:**

The analysis revealed two dense networks characterized by multiple complex connections. In the overall network, symptoms are clustered into psycho‐emotional and cognitive communities, with attention deficits serving as a crucial link between them. One finding was that self‐reported cognitive impairments do not align with objectively measured deficits. Within the mild TBI subgroup, PTSD emerges as a central node in the network.

**Conclusion:**

Network analysis reveals the multidimensional and reciprocal nature of long‐term TBI symptoms. Attention deficits bridge cognitive and psycho‐emotional areas, whereas psycho‐emotional symptoms influence self‐perceived performance. Self‐reported cognitive impairments should be emphasized in therapy as they are linked rather to sleep, visual disturbances, and anxiety than to objective deficits. Network analysis is valuable for understanding TBI symptom complexity and exploring treatment options. Future research should utilize longitudinal designs to validate our findings.

## Graphical abstract

We analyzed persistent symptoms in 250 TBI patients using partial correlation networks. The main findings show that attention deficits link cognitive and psycho‐emotional domains, whereas psycho‐emotional symptoms greatly impact self‐perceived performance. We suggest network analysis as an effective method for understanding the complexity of TBI symptoms and identifying potential treatment options.

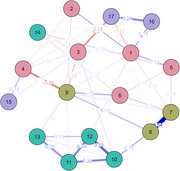

## 1 Introduction/Background

Traumatic brain injury (TBI) is a leading cause of morbidity and mortality affecting more than 50 million people each year worldwide (Maas et al. 2017). TBI sequelae vary widely, encompassing cognitive, behavioral, sensorimotor, and psychological disorders (Pavlovic et al. 2019). TBI is a persistent condition, which poses challenges in standardizing classifications and predicting outcomes due to its variable nature and individual differences in recovery. Pathophysiological assumptions must take into account dynamic, long‐term changes over days, weeks, months, or years (Kenzie et al. 2017; Kenzie et al. 2018) as symptoms following TBI resolve spontaneously, persist, or develop over time.

The wide range of symptoms and enduring consequences following TBI include mental health conditions such as depression and anxiety, cognitive impairment, dizziness, and sleep disorders, among others (Maas et al. 2017). However, numerous studies have illustrated the difficulty in predicting the occurrence and persistence of these symptoms, describing their progression, and providing early prognoses (McMahon et al. 2014; Corrigan et al. 2014; Rickels et al. 2023). This is because symptoms can interact in complex ways often reinforcing or reactivating each other. Hence, the persistent symptoms following TBI may not always directly stem from the initial injury. The symptom complex can be understood as a dynamic, interconnected construct influenced by various factors.

Especially in mild traumatic brain injury (mTBI) cases, the severity of clinical symptoms often surpasses the apparent level of damage. Numerous studies have sought to elucidate the causes, incidence, and severity of symptoms following mTBI. To explain this phenomenon, often referred to as post‐concussion syndrome (PCS), contextual factors such as social and personality influences (King and Kirwilliam 2013; Tellier et al. 2009) are thought to contribute. However, the underlying causes remain largely unexplained.

Thus, the present study employs a distinct approach with the main goal of investigating the intricate relationships and dynamic interactions among long‐term TBI symptoms. Network analysis is a promising and powerful method that has been increasingly used in the field of pathopsychological research in recent years (Fried and Cramer 2017). The psychological network approach conceptualizes mental disorders as complex systems with interacting symptoms (Borsboom 2022) and is able to overcome the classical, diagnostic cause–symptom approach but instead takes into account the heterogeneity as well as potential comorbidities of mental disorders (Fried and Nesse 2015). For example, depression, sleep disturbances, and impaired brain function may not necessarily be direct consequences of an accident (common cause). Rather, depression is closely linked to sleep disturbances and psychomotor slowing, both of which, in turn, affect brain function, concentration, and memory (Fried and Nesse 2015; Borsboom et al. 2011).

Mapping symptoms in networks can reveal information of possible causal relationships, providing insights for both research and clinical practice. However, only few studies used network analysis to investigate the long‐term sequelae of TBI (Fonda et al. 2022; Carmichael et al. 2023; Eagle et al. 2023). Carmichael et al. found that *worrying* and *having difficulty relaxing* may particularly be important symptoms in the maintenance and comorbidity of anxiety and depression following TBI. They identified potential bridges between worrying thoughts and hyperarousal symptoms, as well as between difficulty relaxing and depressive symptoms (Carmichael et al. 2023). Eagle et al. published promising network analysis results from a study of 1500 patients reporting post‐concussion symptoms at multiple time points (Eagle et al. 2023). They concluded that emotional and cognitive symptoms might play a crucial role in predicting long‐term function and quality of life in these patients.

However, these studies predominantly utilized self‐reported data from questionnaires. The aim of the current study was to explore the interrelationships among long‐term symptoms by including objective assessments of neurocognitive functions across a number of neuropsychological domains in a sample with a broad range of TBI severity levels. Furthermore, we accounted for several contextual factors (age, concomitant injuries, and the timespan since the accident) and integrated these into the network models. The following research question was addressed:

1. How are psycho‐emotional, physical, and cognitive symptoms after TBI related to each other?

Due to the distinctive characteristics of mild TBI, this group is examined separately in a second analysis to draw potential conclusions about symptom patterns in PCS:

1. How does the network change when only mildly affected patients are included in the analysis (mTBI subgroup)?

## 2 Methods

### 2.1 Design and Sample

A secondary data analysis was carried out using cross‐sectional routine data from a German trauma center. Ethical approval was granted by the ethics committee of the Bochum University of Applied Health Sciences (K230607_Schuler). The study focused on individuals who had sustained a TBI and continued to experience persistent symptoms for more than 3 months post‐accident. The sample was selected from participants in the Brain Check program (BC) at BG Klinikum Duisburg between January 2017 and December 2022 and included all BC participants with a TBI (ICD‐10 codes S.06xx and/or F.07xx). This program, covered by German statutory accident insurance, aims to prevent chronic progression at an early stage and implements targeted interventions. The timing of the BC after the accident varies for each patient. Healthcare professionals assign patients to BC when they observe complications or deviations in the expected recovery process, such as unusually prolonged symptoms. In BC, patients undergo a comprehensive 10‐day inpatient diagnostic assessment, during which extensive data are collected and recommendations are provided to patients and health care providers.

### 2.2 Measurements

One of the authors (Helen Bindels) systematically extracted data from digital diagnostic and discharge documents and organized them for subsequent statistical analysis. In addition to clinical and sociodemographic variables, Glasgow Coma Scale (GCS) values were retrieved from the emergency doctor's reports to classify TBI severity and specify the mTBI subgroup (GCS 13–15). Factors such as age, latency, and concomitant injuries were considered as covariates in the analysis. Extracranial concomitant injuries related to the accident were documented and classified using the Injury Severity Score (ISS) (Baker et al. 1974). They are common among TBI patients and significantly affect both the manifestation of symptoms and subsequent treatment (Yue et al. 2020). The term “latency” refers to the time interval between the accident and BC. Latency is crucial for understanding the potential long‐term nature of these symptoms, especially since not all patients were assessed at the same time point after the accident. Age is significant across various domains of brain function and social aspects. Furthermore, the analysis included the following symptoms:

#### 2.2.1 Somatic Symptoms

Headache, back pain (cervical and/or lumbar), dizziness (i.e., vertigo, loss of balance), tinnitus, sleep disturbance (all kinds) and visual disturbance (i.e., double vision, blurred vision) were recorded as physical symptoms in semi‐structured interprofessional interviews at the beginning of the BC. All variables were coded as dichotomous (present/not present) regardless of the onset, course, duration, localization, and intensity of the respective symptoms.

#### 2.2.2 Psychological Symptoms

The Hospital Anxiety and Depression Scale (HADS) was used to assess depression‐ and anxiety‐related symptoms (Snaith et al. 1995) on two scales, each ranging from 0 to 21, with higher values indicating more severe symptoms. Presence of post‐traumatic stress disorder (PTSD) was diagnosed by a clinical psychologist.

#### 2.2.3 Cognitive Symptoms

Attentional deficits (“attention”) were evaluated using the Test Battery for Attentional Performance (TAP), assessing alertness, selective and divided attention, and visual scanning (Zimmermann and Fimm 1995). Learning and memory deficits (“memory”) are based on the Verbal Learning and Memory Test (VLMT; Helmstaedter and Durwen 1990), the Visual and Verbal Memory Test (VVM; Schächtele and Schelling 2009), and the forward recall subtest of the Hamburger–Wechsler Intelligence Test for Adults (HA‐WIE‐R; Tewes and Wechsler 1991). Impairments of executive brain function (“executive function”) were assessed by the behavioral assessment of dysexecutive syndrome (BADS; Wilson et al. n.d.) adapted German version 2000), the Standardized Link Probe (SLP; Metzler 2000) and the flexibility subtest of the TAP. Visual perception deficits (“visual perception”; visual construction skills, object recognition) were assessed using subtests of the Visual Object and Space Perception Battery (VOSP; Warrington and James 1992), the Rey Complex Figure Test and Recognition Trial (RCFT; Merten and Blaskewitz 2008) and the Mosaic Test of the HAWIE‐R (Tewes and Wechsler 1991). Each symptom was rated across six levels, from severely impaired to above average, based on age–norm values adjusted for ages and estimated premorbid performance levels. In addition, self‐reported symptoms of concentration difficulties and cognitive deficits (i.e., forgetfulness, word finding difficulties) were assessed during the interview. These symptoms were combined due to high correlation (*r* = 0.68) into a single variable, “subjective cognitive impairment,” indicating that patients experienced either none, one, or both of these issues.

#### 2.2.4 Statistical Analysis

First bivariate Spearman rank correlation coefficients of all variables were calculated. Significance testing was conducted at *α* = 0.05. Subsequently, two regularized partial correlation networks (Epskamp and Fried 2018) were estimated, one with the whole sample and one with mTBI subgroup only. In these networks, symptom variables are depicted as nodes, whereas edges represent relationships between variables (i.e., partial correlations adjusted for all other variables in the network). A detailed description of the network analysis method is provided in the Supporting Information. The hyperparameter ɣ was set to 0.25 (Van Borkulo et al. 2014). Missing values were excluded pairwise for EBICglasso estimation. To determine the importance of each network variable, the centrality indices *strength/degree* and *expected influence (EI)* were computed. The stability of the centrality indices was checked using the correlation stability (CS) coefficient. The weight of the edges is abbreviated to “g” in this paper. All data management was performed with Microsoft Excel (version 2306, 2019), all calculations with the statistical program JASP Team (2023).

## 3 Results

Out of 260 cases identified, 10 were excluded due to early discharge (*n* = 5), another underlying condition (*n* = 3), or a minimally conscious state (*n* = 2). The remaining *n* = 250 were included in the statistical analyses. In the predominantly male sample (78%), 79.1% had suffered mild, 4.9% moderate, and 16.0% severe TBI. The mean age at the time of the accident was 43.1 (± 15.9) years. The mTBI subgroup differs only slightly in most characteristics (see Tables 1 and 2). Only the latency period differs significantly: *M* = 30.2 (± 70.0; Median = 9.0) months for the total sample versus *M* = 14.0 (± 25.5; Median = 6.82) months for mTBI.

**TABLE 1:** Sample characteristics.

	**Total sample**		**mTBI subgroup**	
	***N***	**M (SD)**	***N***	**M (SD)**
**Age**	250	43.1 (± 15.9)	178	45.9 (± 14.5)
**Latency** Time span accident—brain check (months)	250	30.2 (± 70.0) median: 9	178	14.0 (± 25.5) median: 6.82
	***N***	**%**	***N***	**%**
**Sex**				
Men	194	77.8	138	77.5
Women	56	22.4	40	22.5
**Cause of accident**				
Traffic accident	104	41.6	63	35.4
Fall from > 3 m	54	21.6	40	22.5
Simple fall	35	14	31	17.4
Violence	13	5.2	9	5.1
Head trauma	13	5.2	13	7.3
Hit by object	22	8.8	18	10.1
Other/not known	9	3.2	4	2.2
**Length of hospital stay (days)**	250	11.1 (± 8.7)	178	8.2 (± 8.1)
**Concomitant injuries**	(249)			
None	71	28.4	63	35.4
At least one	97	38.8	84	47.2
Polytrauma	81	32.4	31	17.4
**Highest level of education**	(207)		(147)	
No qualification	25	12	19	12.9
Secondary school	123	59.5	89	60.5
University entrance qualification or higher	59	28.5	39	26.5
**Occupational sector**	(228)		(167)	
Blue‐collar worker	140	61.4	107	64.1
White‐collar worker	35	15.4	22	13.2
Other	53	23.2	38	22.7
**Pre‐existing mental disorder**—yes	60	24	46	25.8

**TABLE 2:** Symptom distribution in sample.

	**Total sample**		**mTBI subgroup**	
**Somatic symptoms**	***N***	**%**	***N***	**%**
Headache	141	56.4	113	63.5
Dizziness	103	41.2	76	42.7
Back pain	80	32.0	57	32.0
Sleep disturbance	84	33.6	50	33.7
Vision disturbance	45	18.0	29	16.3
Tinnitus	26	10.4	20	11.2
**Psycho‐emotional symptoms**	***N***	**M (±SD)/%**	***N***	**M (±SD)/%**
Anxiety	222	7.94 (±5.03)	165	7.84 (±5.0)
Depression	223	8.25 (±5.6)	166	8.2 (±5.6)
PTSD	48	19.2	28	15.7
**Cognitive deficits**	***N***	**%**	***N***	**%**
Reported cognitive deficits	142	56.8	99	55.6
Reported concentration difficulties	118	47.2	83	46.6
Attention	165	69.0	114	64.0
Executive function	152	63.3	103	57.9
Memory	166	69.2	113	63.5
Visual perception	58	24.6	35	19.7

Table 3 shows Spearman correlation coefficients between the network variables. Highest correlations were observed for psycho‐emotional and neurocognitive symptoms, such as anxiety and depression (*r* = 0.726, *p* < 0.001), and executive function and memory deficits (*r* = 0.538, *p* < 0.001). Symptoms from different areas were moderately related, with highest correlations found for sleep disturbances with anxiety (*r* = 0.288, *p* < 0.001) and PTSD (*r* = 0.251, *p* < 0.001). Correlation coefficients in the total sample and the mTBI subgroup differed only marginally.

**TABLE 3:** Bivariate Spearman Correlation of all variables included in *N*1 (total sample; left side) and *N*2 (mTBI sample; right side).

Variable	1	2	3	4	5	6	7	8	9	10	11	12	13	14	15	16	17
1. Headache	—	**0.170**	0.112	−0.026	0.145	−0.035	**0.215**	**0.186**	0.081	0.127	0.116	**0.150**	−0.044	−0.076	−0.121	0.063	**−0.169**
2. Back pain	**0.168**	—	0.040	−0.092	−0.042	0.055	0.129	0.104	0.141	0.035	0.040	0.080	0.071	−0.089	−0.052	−0.045	0.075
3. Dizziness	0.110	0.033	—	0.124	0.019	−0.102	−0.088	−0.004	**−0.163**	**0.155**	0.057	0.001	−0.053	**−0.156**	**0.181**	−0.128	**−0.269**
4. Tinnitus	0.034	−0.010	**0.193**	—	−0.012	0.053	−0.094	−0.067	0.112	−0.001	−0.002	−0.017	−0.006	−0.009	0.144	0.088	−0.107
5. Visual disturbance	0.074	−0.055	0.029	0.010	—	−0.115	**0.176**	0.079	−0.068	−0.007	−0.077	−0.006	−0.037	0.131	−0.019	−0.047	−0.118
6. Sleep disturbance	0.019	0.075	−0.087	0.060	−0.046	—	**0.259**	**0.191**	**0.259**	0.076	0.072	−0.009	0.015	**0.166**	0.042	0.102	0.132
7. Anxiety	**0.187**	0.119	−0.071	−0.062	**0.183**	**0.288**	—	**0.725**	**0.224**	**0.177**	0.081	**0.168**	0.007	**0.164**	**−0.159**	0.085	0.122
8. Depression	**0.187**	0.084	−0.035	−0.071	**0.144**	**0.235**	**0.726**	—	**0.287**	**0.297**	**0.156**	**0.171**	0.079	0.114	**−0.194**	**0.176**	**0.182**
9. PTSD	0.044	**0.129**	**−0.129**	−0.075	−0.017	**0.251**	0.319	**0.304**	—	−0.001	**0.168**	0.067	0.120	0.025	−0.069	0.051	0.085
10. Attention	0.108	0.003	**0.174**	−0.028	0.003	0.059	**0.126**	**0.234**	−0.083	—	**0.433**	**0.498**	**0.234**	−0.001	−0.023	**0.183**	−0.026
11. Memory	0.078	0.022	0.046	−0.049	−0.100	0.118	0.079	**0.148**	0.127	**0.417**	—	**0.499**	**0.342**	−0.010	0.026	0.064	0.026
12. Executive function	0.111	0.051	−0.006	−0.047	−0.020	0.023	**0.133**	**0.149**	0.063	**0.480**	**0.538**	—	**0.253**	−0.007	−0.024	0.087	0.034
13. Visual perception	−0.014	0.039	−0.027	−0.035	0.046	0.042	0.033	0.095	0.066	**0.307**	**0.335**	**0.307**	—	0.050	0.081	0.056	0.084
14. Subgroup cognitive impairment	−0.079	−0.087	**−0.179**	−0.047	0.124	**0.213**	**0.198**	**0.161**	0.079	0.033	0.062	0.072	0.099	—	**−0.152**	−0.003	−0.018
15. Age	0.016	0.002	**0.233**	0.152	−0.053	0.023	−0.164	**−0.168**	−0.125	−0.033	−0.051	−0.066	−0.017	**−0.176**	—	0.060	0.049
16. Latency	−0.018	−0.042	**−0.132**	−0.006	−0.040	0.068	0.110	**0.192**	0.096	**0.202**	**0.133**	**0.167**	0.117	0.036	**−0.177**	—	**0.255**
17. Concomitant injuries	**−0.220**	0.034	**−0.184**	−0.101	−0.005	0.109	0.112	**0.157**	0.085	0.011	0.102	0.108	**0.140**	−0.001	**−0.133**	**0.378**	—

*Note*: Significant correlations are bold printed.

**FIGURE 1:**
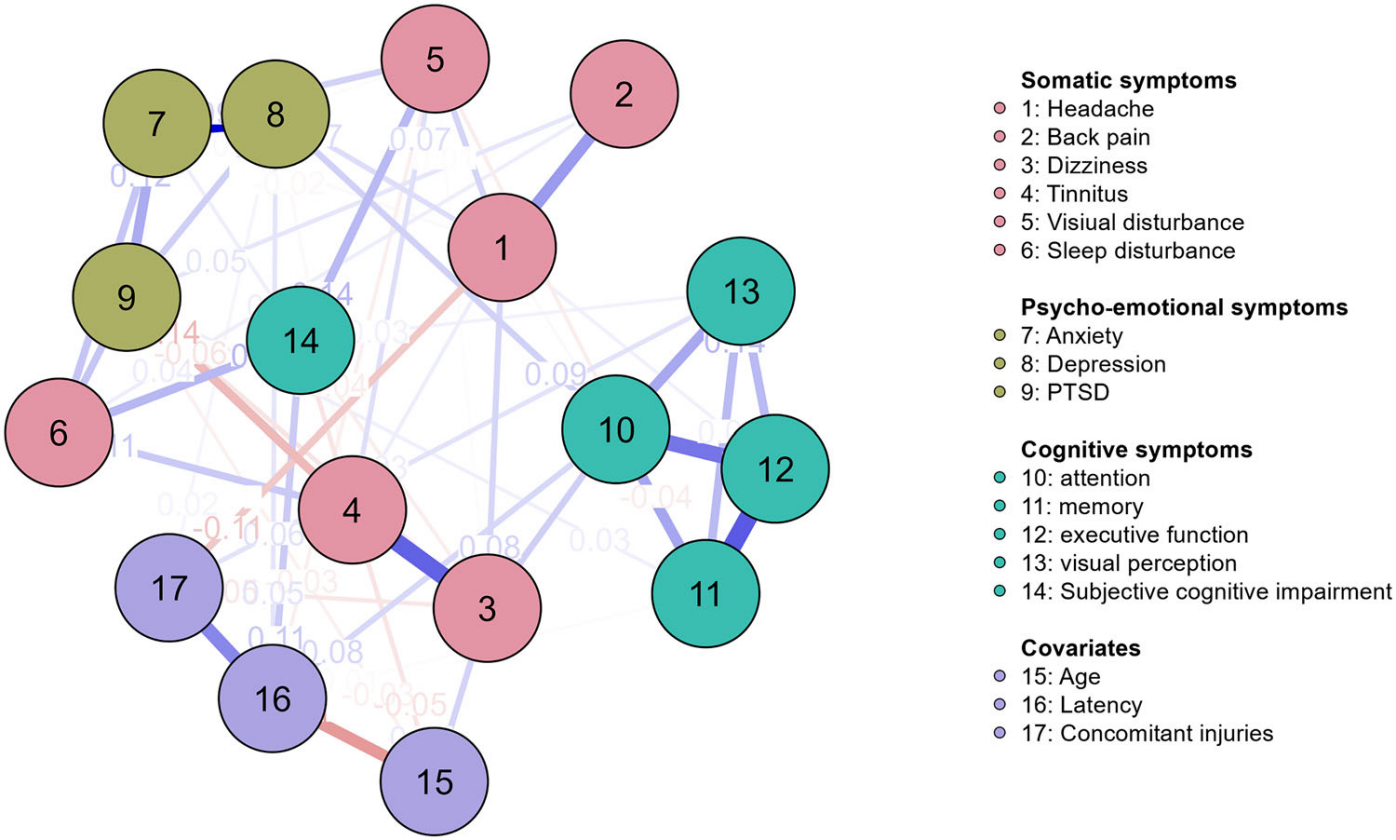
Network (1) correlations of psycho‐emotional, physical, and cognitive symptoms in the total sample.

Figure 1 shows network N1 estimated for all cases (*N* = 250) including 14 symptoms and three covariates. In this network, 56 of the possible 136 edges are presented, which corresponds to a proportion of 41.2% and means a sparsity of 58.8%. The respective edge weights can be found in the Supporting Information. The strongest independent associations are between anxiety and depression (*g* = 0.534), memory and executive function (*g* = 0.345), dizziness and tinnitus (*g* = 0.329), and headaches and back pain (*g* = 0.209). As already shown in the correlation matrix (Table 1), there is only a slight association between objectively measured cognitive deficits and subjectively reported ones, with a marginal correlation noted specifically for visual perception (*g* = 0.027).

The centrality indices (Supporting Information) can be seen as indicators of the importance of a node in the overall network. For N1, anxiety is both the strongest node in the network and the one with the highest influence. In second place for both indices is the node attention.

The two stability plots (Supporting Information) indicate that N1 may be unstable and should therefore be interpreted with caution. Within the case drop bootstrap analysis, the mean values of the newly generated strength values remain approximately at a correlation of cor = 0.7 with the original sample, even when the sample size is reduced by 25%. However, their confidence intervals significantly diverge. The CS coefficients of all indices are below 0.05 which means low overall stability.

**FIGURE 2:**
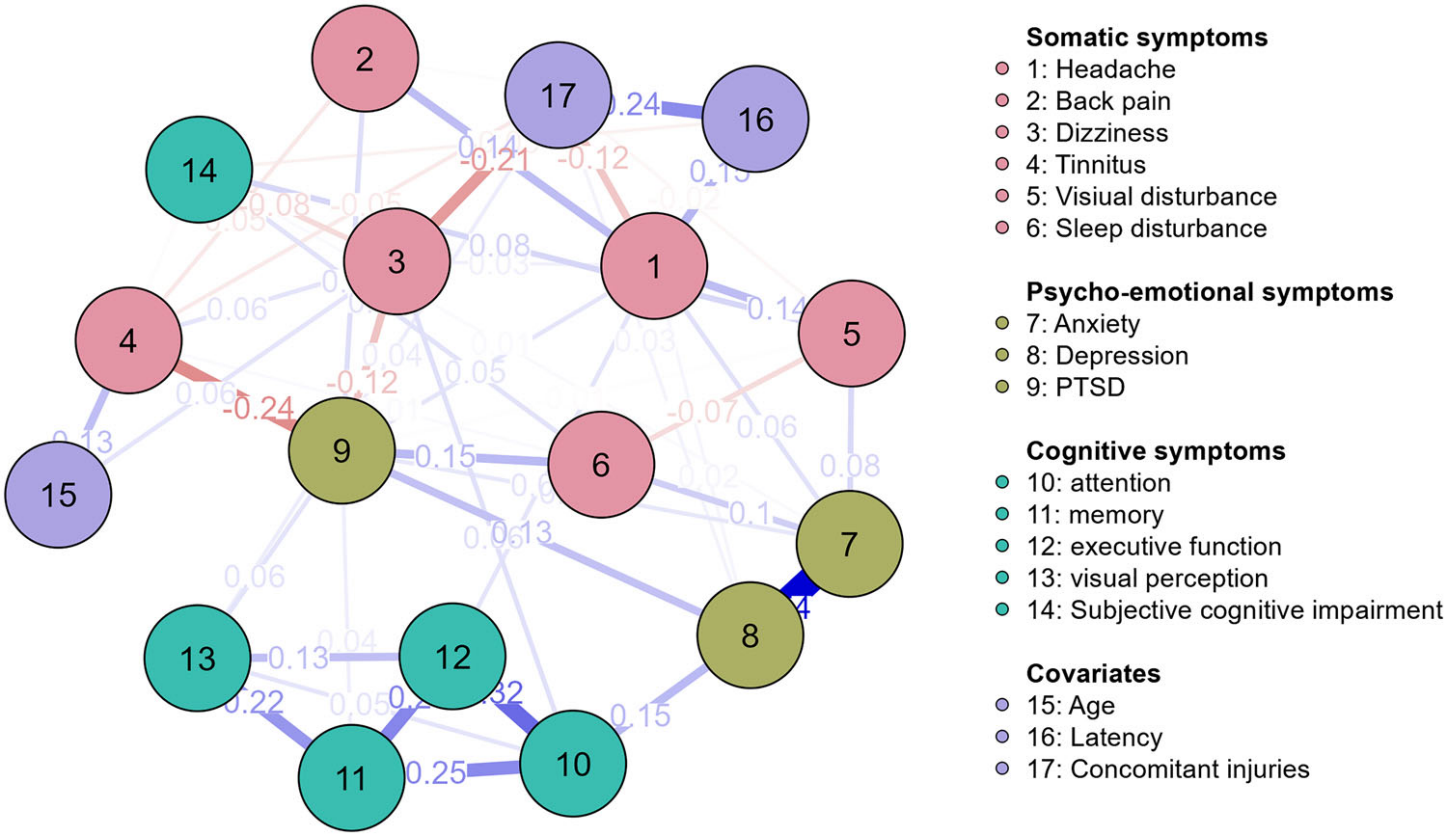
Network (2) correlations of psycho‐emotional, physical, and cognitive symptoms in the mTBI subgroup.

Figure 2 shows the network N2 estimated exclusively for the mTBI subgroup (GCS 13–15; *n* = 178). In N2, 48 out of 136 possible edges were included, corresponding to 35.3%. Compared to N1, this model holds a higher sparsity (64.7%). Similar to N1, the covariates are placed on the outer edge by the algorithm and have little interaction with the symptoms themselves. However, within this subgroup, patients with less concomitant injuries are more likely to report headaches (*g* = −0.118) and dizziness (*g* = −0.209). PTSD, depression, and anxiety are identified as the strongest nodes, with the latter two also exhibiting the highest EI on network dynamics. Again, the stability graphs indicate low stability.

## 4 Discussion

In the present study, persistent symptoms after TBI were analyzed within psychometric network models to shed light on the dynamic interaction of these symptoms. This study benefits from interdisciplinary routine data collection, especially comprehensive neuropsychological testing conducted over multiple days. However, as this is a cross‐sectional analysis, the results should be interpreted as an exploratory step for generating causal hypotheses about between‐person symptom relationships, not within‐person dynamics.

The strong associations within the psycho‐emotional symptom domain appear plausible and unsurprising as, for example, depression and anxiety are generally considered as comorbidities. A bidirectional causal influence can be assumed for these symptoms.

The clear grouping of the four neuropsychological cognitive symptoms reflects that people with TBI usually have deficits in several closely interrelated functional areas of the brain (McAllister 2011). In particular, attentional deficits are typically associated with impaired performance in other functional domains (Stierwalt and Murray 2002). In N1, the attention node is the only objectively measured cognitive symptom that shows associations with other symptom domains through its links to depression and dizziness. This suggests that attentional deficits may act as a bridge symptom between the neurocognitive cluster and other symptom areas.

Interestingly, there is no (zero‐order or partial) correlation between self‐reported cognitive deficits and objective cognitive test results. This could mean that patients’ self‐perception or self‐assessment differs from their actual level of performance. This phenomenon has been reported frequently in TBI populations as well as in other neurological conditions (Gass and Apple 1997; Schwartz, Kozora, and Zeng 1996; Spencer et al. 2010). This disparity may stem from a lack of insight into the illness. Conditions such as anosognosia can lead to overestimation of one's cognitive abilities. However, Vos et al. suggest that this explanation alone cannot fully account for the significant gap (Vos et al. 2020). They found that organic brain disorders of self‐perception were more prevalent in moderate and severe TBI. This contrasts with the observed results across all TBI severities. It is widely recognized that attention tests used in neuropsychological assessments do not fully capture the complexity of attention demands experienced in daily life. As a result, everyday challenges may go undetected through objective testing. In addition, patients may distort their memory of premorbid performance often overestimating their functioning before the accident (Gunstad and Suhr 2001). This recall bias can lead to an exaggerated perception of impairment after the injury. Depressed individuals often exhibit a tendency toward negative self‐evaluation, leading to overly negative assessments of cognitive performance. Moreover, subjectively perceived and standardized tested cognitive impairment may follow different assumptions or have different impacts. Patients’ experience of cognitive impairment might primarily be influenced by psycho‐emotional symptoms. Therefore, interventions seeking to improve perceived difficulties should address psychological issues (e.g., coping with illness) rather than focusing solely on cognitive training. For example, therapists could use intraindividual symptom networks to enhance patients’ understanding of how their symptoms interact (Kashihara et al. 2024).

Cognitive deficits may also affect psychological well‐being and might lead to depression by hindering participation in meaningful activities such as work and social interactions (Pagulayan et al. 2008). Thus, an association between depression and the objective cognitive domain was expected in the networks. Bivariate Spearman correlations show links between depression and memory (*r* = 0.148) and executive function (*r* = 0.149). However, in N1, the links between depression and memory/executive function are no longer direct but mediated via the attention node. This underscores the pivotal role of attention in the association between depression and neurocognitive deficits. Further research should explore how different attentional dimensions, such as divided and sustained attention, relate to psycho‐emotional or somatic symptoms. In addition, studying structural and functional connectivity in a dual‐layered network could deepen our understanding of symptom dynamics (Caeyenberghs et al. 2013).

In N1 a longer latency period is directly associated with lower attention functions, higher self‐reported cognitive impairment, and higher depression. This aligns with findings by Scholten et al., who demonstrated increasing depression and anxiety rates over time post‐TBI (Scholten et al. 2016). One possible explanation is the persistence of accident‐related stressors, especially if complications arise, delaying the natural recovery process. Furthermore, longer latency correlates with more concomitant injuries at the time of the accident (proximity to latency period *g* = 0.253; bivariate correlation: *r* = 0.378). Patients with multiple concomitant extracranial injuries often undergo prolonged inpatient and outpatient rehabilitation. However, TBI‐specific symptoms may not be evaluated until the later stages of rehabilitation. The initial focus on “obvious” injuries, such as orthopedic treatment, might lead to a perception of well‐integrated care while potentially overlooking cognitive and psychological consequences. A standard follow‐up appointment, no later than 3 months after the accident (Strowitzki 2018), could help identify persisting symptoms earlier and enable more effective treatment.

### 4.1 mTBI Network

Networks N1 and N2 are similar in most connections. However, some differences are worth discussing:

The PTSD node moves to the center of the network, showing additional links with headaches, back pain, visual perception, and memory problems. There are significant symptom and comorbidity overlap between PTSD and mTBI (i.e., sleep disorders, poor concentration, and pain), making their distinction challenging (Tanev et al. 2014; Simonovic et al. 2023). These findings align with N2, where depression, back pain, and headaches were directly associated with PTSD. MTBI patients often come to BC due to their psycho‐emotional issues, whereas those with moderate to severe injuries may have other primary deficits (e.g., physical limitations and severe cognitive deficits). Thus it is essential in everyday clinical practice to acknowledge that mTBI patients with comorbid PTSD may experience heightened distress and be particularly susceptible to psychological comorbidities (Tanev et al. 2014). They require special attention in diagnosis and treatment, as isolated PTSD treatment approaches may not be equally effective for those with comorbidities (Simonovic et al. 2023).

Furthermore, in N2, headache emerges as a significant component in the interplay of symptoms, as evidenced by the numerous connections compared to N1. Interestingly, this “post‐traumatic headache” is more common in mTBI cases than in severe ones (Maas et al. 2022) as in our sample, 63.5%  versus 34.0%. According to N2, individuals with multiple concomitant injuries are less likely to experience headaches, suggesting that headache is more prevalent among patients with isolated mTBI. This observation aligns with the concept of PCS, which typically characterizes concussions without structural brain damage (Polinder et al. 2018). However, such persistent symptoms associated with PCS are also found in non‐TBI populations, such as chronic pain patients, who often experience sleep disturbances, depression, and irritability (Iverson and McCracken 1997). Prospective cohort studies could clarify the staggered onset and dynamic development of headache, anxiety, and depression after mTBI.

### 4.2 Limitations

This study has several limitations. First, this is a secondary analysis of patient data that were gathered during inpatient hospital stays for diagnostic and therapeutic purposes, not for research. For some symptoms, data were primarily collected through semi‐structured medical history interviews. In these cases, they could only be recorded dichotomously (yes/no) without any further information. Second, comparisons of networks drawn solely based on visual graphics can be misleading and should be interpreted with caution. Although statistical tests such as the “Network Comparison Test” can validate visual comparisons with numerical data (Tantardini et al. 2019), they are not suitable for comparing a total sample with a subgroup, as was done in this study. However, the sample size was too small to estimate complex statistical network models for severe TBI only. Furthermore, the differences observed between the full‐sample and mTBI‐only models may reflect instability rather than true differences between groups. Third, the small sample size likely contributes to the low‐stability parameters. Strong conclusions about causal relationships cannot be drawn from this study without future longitudinal studies with higher sample sizes. Fourth, the sample consists exclusively of persons insured by the German statutory accident insurance, that is, all persons were injured in accidents at work or on the way to work. This partly explains the characteristics of the sample, such as the low average age (working age) and the high proportion of male patients (“blue‐collar workers”). This may also have influenced symptom reporting, such as seeking compensation or disability pensions. Moreover, the sample may not be fully representative of all TBI patients, as it includes only those with persisting symptoms. Future studies should aim to analyze a more diverse sample of TBI patients. Finally, patients differed in the time interval between accident and their assessment in the BC. However, we integrated latency as a covariate in the networks as it is a crucial factor in the potential chronicity of symptoms.

## 5 Conclusion

The analysis of long‐term persistent symptoms after TBI using network analysis highlights the multidimensional and reciprocal nature of TBI consequences. While causal relationships cannot be determined in this cross‐sectional study, the results suggest that psycho‐emotional symptoms are substantially related to other symptoms such as attention and headaches, as well as self‐perception of performance. Attention deficits play a key role in bridging cognitive and psycho‐emotional symptom areas. Self‐reported cognitive impairment often differs from objectively measured deficits, with the former being closely linked to sleep, visual disturbances, and anxiety. Therapy should place more emphasis on perceived cognitive symptoms and their associations rather than solely on objective test results. Our findings also demonstrate that network analysis is a valuable tool both for capturing the complexity of persistent TBI symptoms and for exploring potential treatment options. Future research should accurately differentiate heterogeneous symptoms, such as attention deficits or subjective cognitive disturbances, and use longitudinal designs to examine changes in the complex dynamic symptom network over time.

## Author Contributions

**Helen Bindels**: writing–original draft, conceptualization, investigation, data curation, methodology. **Sascha Sommer**: writing–review and editing, validation. **Tobias Ohmann**: writing–review and editing, supervision. **Susann Seddigh**: writing–review and editing. **Michael Schuler**: project administration, writing–review and editing, methodology, supervision.

## Conflicts of Interest

The authors declare no conflicts of interest.

### Peer Review

The peer review history for this article is available at https://publons.com/publon/10.1002/brb3.70316.

## Supporting information



Supporting Information

## Data Availability

The data that support the findings of this study are available from the corresponding author upon reasonable request.
