# Supplementary material for: How Are TBI Symptoms Interconnected? A Network Analysis Approach
Source: Brain Behav. 2025 Feb 16;15(2):e70316. doi: 10.1002/brb3.70316 (PMC11830752; doi:10.1002/brb3.70316)
Supplement: Supplementary file 1 — Supporting Information [file BRB3-15-e70316-s001.docx]

**Online Supplement A: Detailed Methodology of Network Analysis**

**Psychological networks:** We estimated “psychological networks" following the approach presented by Epskamp and Fried (2018), utilizing regularized and weighted partial correlation networks. In psychological networks, observed variables are represented as nodes (circles) and associations between the nodes are represented as edges (lines). In regularized partial correlation networks, the edges (or edge weights) are partial correlation, i.e. correlations between two nodes after controlling for associations with all other variables in the network. Therefore, edge weights quantify the strength and direction of linear associations, adjusted for potential confounding factors (other nodes). In our analysis, the nodes represent symptoms (e. g. depression or attention) and edge weights represent partial correlations between symptoms (e.g. the linear association between depression and attention, controlling for all other symptoms in the network). Unconnected symptoms (i.e. with edge weight = 0) are conditionally independent given all other symptoms in the network (1) while edge weights unequal zero can be regarded as indicators of a potential causal relationship between the respective nodes (2).

**Graphical Representation:** To arrange of the nodes in the graphical network model, we used the Fruchterman-Reingold algorithm (3), which aims to ensure that edges (lines) are as short as possible and nodes connected by edges are placed close together. Additionally, this approach is sensitive to even the smallest changes (e.g., minor differences in edge weights (4)). As a result, an easily interpretable visualization of the graph is produced. For both networks N1-N2, the option “Do not update Layout” was selected, thereby maintaining the same layout to more clearly highlight differences.

**Regularization:** Due to sample fluctuations, parameter estimates are rarely exactly zero, leading to ‘spurious edges’ (false positives) that can misrepresent the network as they might suggest relationships that are not actually relevant and could complicate the visual interpretation of the network (2). To minimize these, we applied the EBICglasso method (5,6). This method combines the Extended Bayesian Information Criterion (EBIC) with the Graphical Lasso estimator. This method enables the selection of an optimal model that achieves the best balance between fit and sparsity. The sparsity of the network is determined by the proportion of non-existent connections (edge weights = 0). Additionally, the hyperparameter gamma (ɣ), which is manually set—typically between 0 and 0.5—determines the preference for simpler or more complex models (7). When ɣ is set to 0, sparsity is low, and many edges are included in the network, accepting the presence of spurious edges. Conversely, at ɣ = 0.5, edges that are close to 0 are eliminated, which increases the risk of excluding connections that might be relevant in reality. In our study the hyperparameter γ was set to 0.25 (following Van Borkulo, 2014 (8)) to balance the exploratory nature of the analysis with a conservative estimation approach.

**Centrality and stability:** To assess the relevance of individual nodes in the network, two centrality indices were calculated:

1. **Strength/Degree** measures the strength of direct connections to all other symptoms, based on the sum of edge weights. This parameter is one of the fundamental constants in (social) network research, which was directly adopted from other fields during the development of psychopathological networks (9).
2. **Expected Influence (EI)** calculates a node’s cumulative influence on all other variables in the network. The EI parameter was introduced in 2016 by Robinaugh et al. (10) and is considered sensitive even when a network consists of both positive and negative connections, as is often the case in (psychological) symptom studies. Together with the Strength value, it is regarded as one of the most robust indices.

All values are standardized (z-scores) to ensure comparability.

However, centrality indices have been subject to criticism for some time, not least due to their unstable estimations in cross-sectional studies (7,9) Centrality indices were adapted with minor modifications from technical and social network analyses to psychopathological networks. However, questions regarding their added value and substantive relevance within psychopathological network research have increasingly been raised (9). Moreover, low stability of centrality indices may also indicate that edges and nodes have similar strengths, making it difficult to establish a weighted ranking (Burger et al., 2023). Due to these reasons, we calculated the centrality indices in the present study but used them only as supplementary elements for interpretation.

Network stability indicates that moderate sample changes do not lead to major result shifts in centrality indices. The Correlation Stability coefficient (CS) indicates the maximum percentage of cases that can be removed from a sample (casedrop bootstrap) in order to obtain a predetermined correlation - cor=0.7 by default - between the indices of the original sample and those of the recalculated reduced samples with a 95% probability. A CS coefficient of at least 0.25, ideally 0.5, is recommended for adequate stability (7). Following Epskamp et al. (2017) (11), stability was tested using nonparametric bootstrapping with 1000 iterative resamples to produce 95% confidence intervals (CIs) for edge weights and centrality indices. These CIs reflect the precision of estimates rather than significance tests against zero.

**References**:

1. Borsboom D, Deserno MK, Rhemtulla M, Epskamp S, Fried EI, McNally RJ, u. a. Network analysis of multivariate data in psychological science. Nat Rev Methods Primer. 19. August 2021;1(1):58.

2. Epskamp S, Fried EI. A tutorial on regularized partial correlation networks. Psychol Methods. Dezember 2018;23(4):617–34.

3. Fruchterman TMJ, Reingold EM. Graph drawing by force‐directed placement. Softw Pract Exp. November 1991;21(11):1129–64.

4. Burger J, Andikkhash V, Jager N, Anderbro T, Blanken TF, Klintwall L. A novel approach for constructing personalized networks from longitudinal perceived causal relations. Behav Res Ther. 11. Dezember 2023;173:104456.

5. Foygel R, Drton M. Extended Bayesian Information Criteria for Gaussian Graphical Models. ArXiv Pre-Print Serv [Internet]. 30. November 2010; Verfügbar unter: https://arxiv.org/abs/1011.6640

6. Friedman J, Hastie T, Tibshirani R. Sparse inverse covariance estimation with the graphical lasso. Biostatistics. 1. Juli 2008;9(3):432–41.

7. Epskamp S, Borsboom D, Fried EI. Estimating psychological networks and their accuracy: A tutorial paper. Behav Res Methods. Februar 2018;50(1):195–212.

8. Van Borkulo CD, Borsboom D, Epskamp S, Blanken TF, Boschloo L, Schoevers RA, u. a. A new method for constructing networks from binary data. Sci Rep. 1. August 2014;4(1):5918.

9. Bringmann LF, Elmer T, Epskamp S, Krause RW, Schoch D, Wichers M, u. a. What do centrality measures measure in psychological networks? J Abnorm Psychol. November 2019;128(8):892–903.

10. Robinaugh DJ, Millner AJ, McNally RJ. Identifying highly influential nodes in the complicated grief network. J Abnorm Psychol. August 2016;125(6):747–57.

11. Epskamp S, Kruis J, Marsman M. Estimating psychopathological networks: Be careful what you wish for. Marinazzo D, Herausgeber. PLOS ONE. 23. Juni 2017;12(6):e0179891.

**ONLINE SUPPLEMENT B – Additional results**

| **Variable** | **1** | **2** | **3** | **4** | **5** | **6** | **7** | **8** | **9** | **10** | **11** | **12** | **13** | **14** | **15** | **16** | **17** |
| --- | --- | --- | --- | --- | --- | --- | --- | --- | --- | --- | --- | --- | --- | --- | --- | --- | --- |
| 1. **Headache** | 0.000 | 0.138 | 0.030 | 0.000 | 0.138 | 0.000 | 0.061 | 0.021 | 0.052 | 0.000 | 0.000 | 0.054 | 0.000 | 0.000 | 0.000 | 0.154 | -0.118 |
| 1. **Back pain** | 0.209 | 0.000 | 0.000 | -0.047 | 0.000 | 0.000 | 0.000 | 0.000 | 0.067 | 0.000 | 0.000 | 0.000 | 0.000 | 0.000 | 0.000 | -0.010 | 0.000 |
| 1. **Dizziness** | 0.085 | 0.000 | 0.000 | 0.055 | 0.000 | 0.000 | 0.000 | 0.000 | -0.124 | 0.063 | 0.000 | 0.000 | 0.000 | -0.077 | 0.055 | 0.000 | -0.209 |
| 1. **Tinnitus** | 0.035 | 0.000 | 0.329 | 0.000 | 0.000 | 0.012 | 0.000 | 0.000 | -0.241 | 0.000 | 0.000 | 0.000 | 0.000 | 0.000 | 0.135 | 0.000 | -0.045 |
| 1. **Visual disturbances** | 0.087 | 0.000 | 0.007 | 0.065 | 0.000 | -0.075 | 0.081 | 0.000 | -0.009 | 0.000 | 0.000 | 0.000 | 0.000 | 0.085 | 0.000 | 0.000 | -0.020 |
| 1. **Sleep**   **disturbances** | 0.000 | 0.035 | 0.000 | 0.111 | 0.000 | 0.000 | 0.101 | 0.000 | 0.147 | 0.000 | 0.000 | 0.000 | 0.000 | 0.060 | 0.000 | 0.000 | 0.000 |
| 1. **Anxiety** | 0.012 | 0.000 | 0.000 | 0.000 | 0.091 | 0.119 | 0.000 | 0.540 | 0.048 | 0.000 | 0.000 | 0.000 | 0.000 | 0.014 | 0.000 | 0.000 | 0.000 |
| 1. **Depression** | 0.069 | 0.000 | 0.000 | -0.015 | 0.009 | 0.002 | 0.534 | 0.000 | 0.129 | 0.145 | 0.000 | 0.000 | 0.000 | 0.000 | 0.000 | 0.000 | 0.026 |
| 1. **PTSD** | 0.000 | 0.049 | -0.055 | -0.144 | 0.000 | 0.116 | 0.175 | 0.099 | 0.000 | 0.000 | 0.036 | 0.000 | 0.056 | 0.000 | 0.000 | 0.000 | 0.000 |
| 1. **Attention** | 0.012 | 0.000 | 0.099 | 0.000 | 0.000 | 0.000 | 0.000 | 0.093 | 0.000 | 0.000 | 0.247 | 0.316 | 0.054 | 0.000 | 0.000 | 0.000 | 0.000 |
| 1. **Memory** | 0.000 | 0.000 | 0.000 | 0.000 | -0.036 | 0.000 | 0.000 | 0.000 | 0.031 | 0.184 | 0.000 | 0.257 | 0.220 | 0.000 | 0.000 | 0.000 | 0.000 |
| 1. **Executive function** | 0.029 | 0.000 | 0.000 | 0.000 | 0.000 | 0.000 | 0.000 | 0.000 | 0.000 | 0.287 | 0.345 | 0.000 | 0.134 | 0.000 | 0.000 | 0.000 | 0.000 |
| 1. **Visual perception** | 0.000 | 0.000 | 0.000 | 0.000 | 0.000 | 0.000 | 0.000 | 0.000 | 0.000 | 0.181 | 0.141 | 0.145 | 0.000 | 0.000 | 0.000 | 0.000 | 0.038 |
| 1. **Sub. Cog. impairment** | 0.000 | 0.000 | -0.036 | 0.000 | 0.144 | 0.148 | 0.034 | 0.000 | 0.000 | 0.000 | 0.000 | 0.000 | 0.027 | 0.000 | -0.010 | -0.030 | 0.000 |
| 1. **Age** | 0.000 | 0.000 | 0.091 | 0.040 | 0.000 | 0.000 | 0.000 | 0.000 | -0.029 | 0.000 | 0.000 | 0.000 | 0.000 | -0.054 | 0.000 | 0.000 | 0.000 |
| 1. **Latency** | 0.000 | 0.000 | 0.000 | 0.000 | -0.033 | 0.000 | 0.000 | 0.055 | 0.000 | 0.082 | 0.008 | 0.000 | 0.000 | 0.109 | -0.212 | 0.000 | 0.244 |
| 1. **Concomitant injuries** | -0.113 | 0.003 | -0.048 | 0.000 | 0.000 | 0.000 | 0.000 | 0.021 | 0.000 | 0.000 | 0.000 | 0.000 | 0.058 | 0.000 | 0.000 | 0.250 | 0.000 |

Online Supplement Table 1: Network Weights N1 (left side) & N2 (right side)


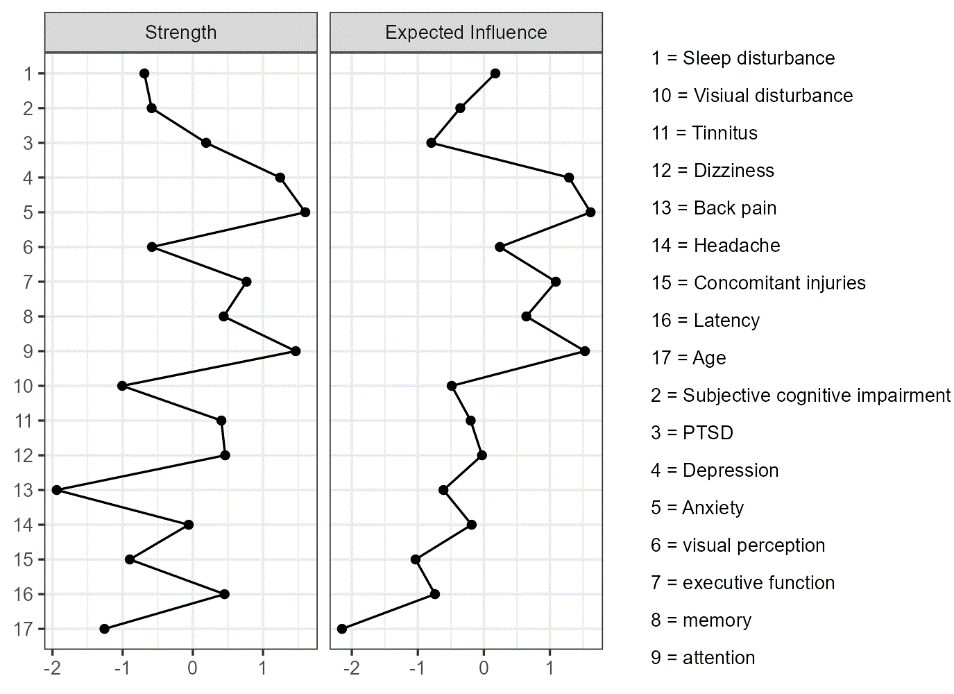


Online Supplement Figure 1: Centrality Plot N1


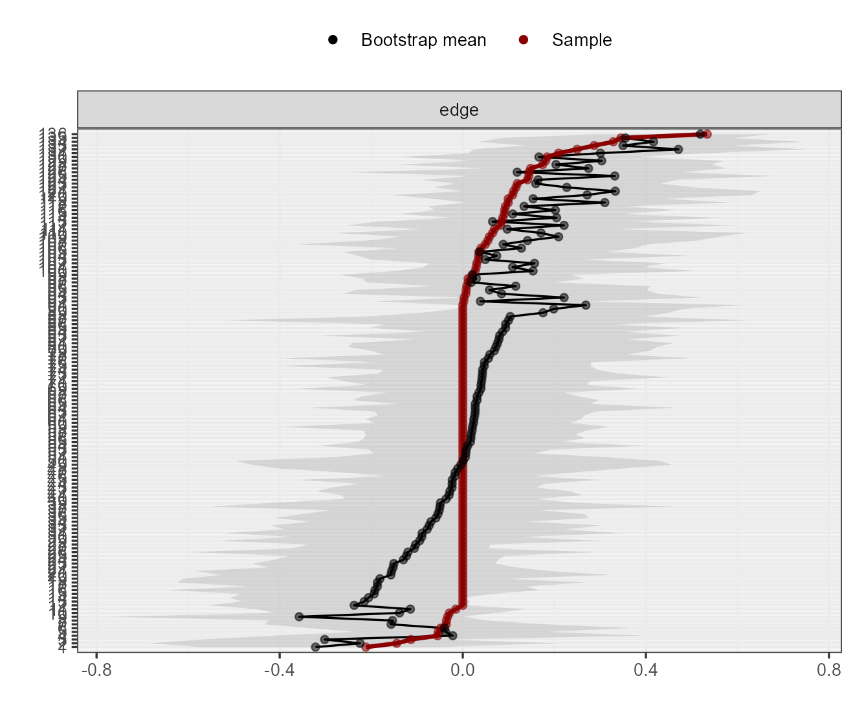


Online Supplement Figure 2: Bootstrap Edges N1


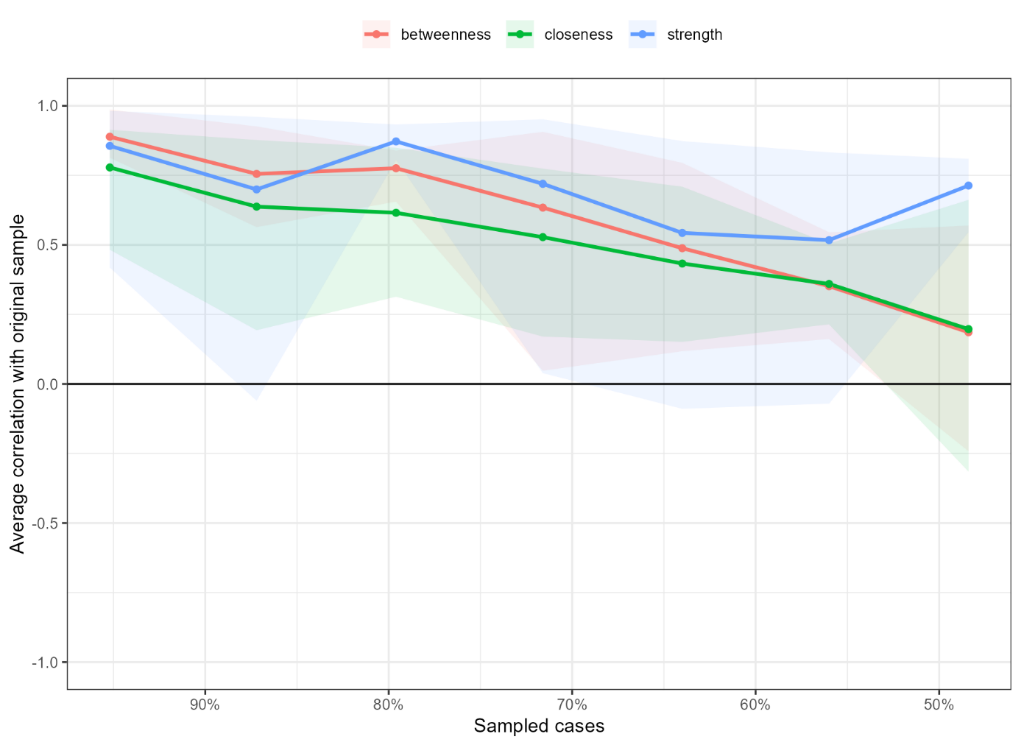


Online Supplement Figure 3: Casedrop Bootstrap Stability N1


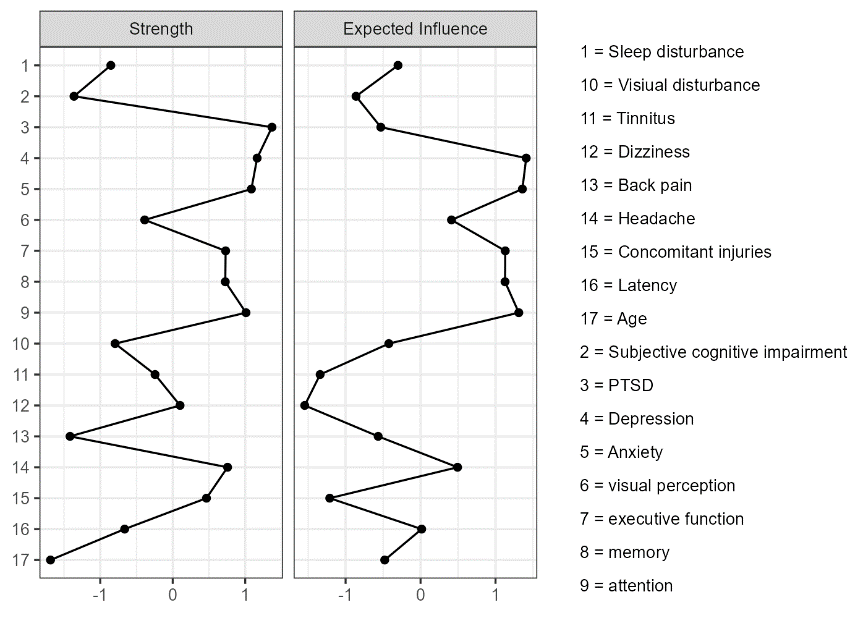


Online Supplement Figure 4: Centrality plot N2


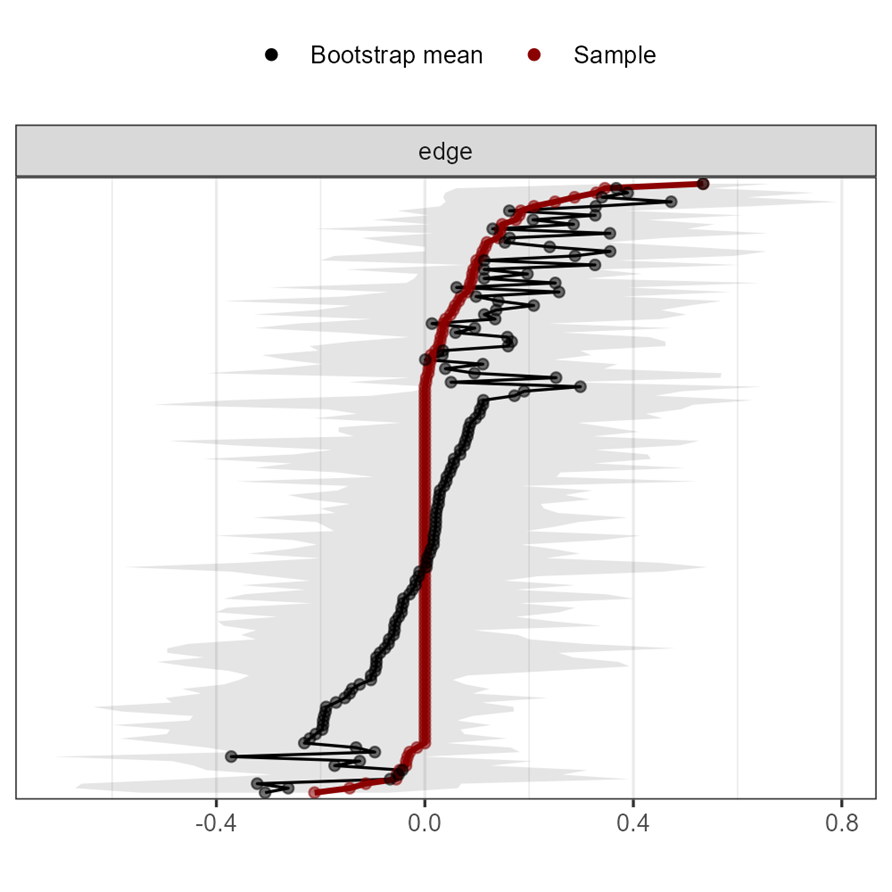


Online Supplement Figure 5: Boostrap Edges N2


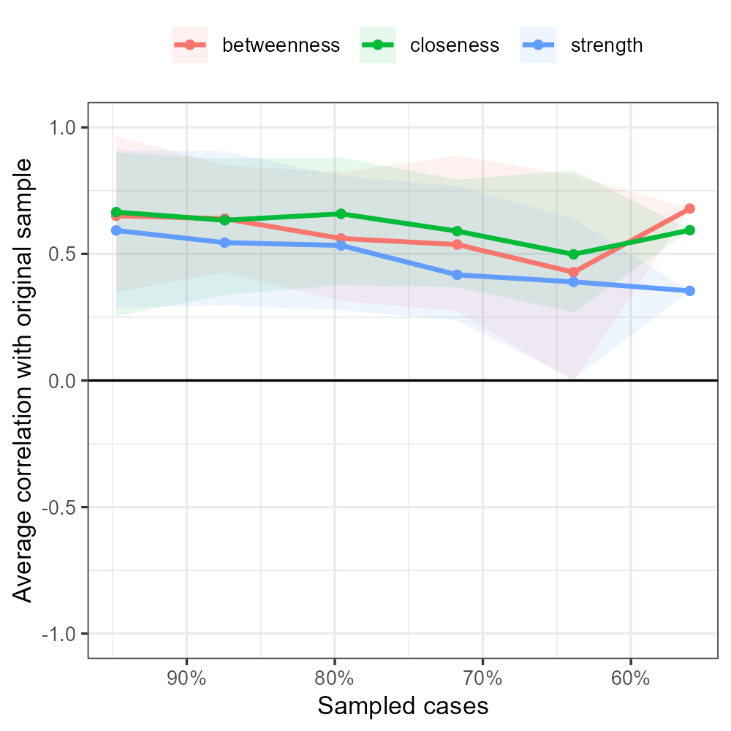


Online Supplement Figure 6: Casedrop Bootstrap Stability N2
